# Serine- and Threonine/Valine-Dependent Activation of PDK and Tor Orthologs Converge on Sch9 to Promote Aging

**DOI:** 10.1371/journal.pgen.1004113

**Published:** 2014-02-06

**Authors:** Mario G. Mirisola, Giusi Taormina, Paola Fabrizio, Min Wei, Jia Hu, Valter D. Longo

**Affiliations:** 1 Dipartimento di Biotecnologie Mediche e Forensi (DiBiMeF) Università di Palermo, Palermo, Italy; 2 Laboratoire de Biologie Moléculaire de la Cellule, Centre National de la Recherche Scientifique, Ecole Normale Supérieure de Lyon, Université de Lyon, Lyon, France; 3 Longevity Institute and Dept. of Biological Sciences School of Gerontology, University of Southern California, Los Angeles, California, United States of America; Stanford University Medical Center, United States of America

## Abstract

Dietary restriction extends longevity in organisms ranging from bacteria to mice and protects primates from a variety of diseases, but the contribution of each dietary component to aging is poorly understood. Here we demonstrate that glucose and specific amino acids promote stress sensitization and aging through the differential activation of the Ras/cAMP/PKA, PKH1/2 and Tor/S6K pathways. Whereas glucose sensitized cells through a Ras-dependent mechanism, threonine and valine promoted cellular sensitization and aging primarily by activating the Tor/S6K pathway and serine promoted sensitization via PDK1 orthologs Pkh1/2. Serine, threonine and valine activated a signaling network in which Sch9 integrates TORC1 and Pkh signaling via phosphorylation of threonines 570 and 737 and promoted intracellular relocalization and transcriptional inhibition of the stress resistance protein kinase Rim15. Because of the conserved pro-aging role of nutrient and growth signaling pathways in higher eukaryotes, these results raise the possibility that similar mechanisms contribute to aging in mammals.

## Author Summary

Calorie restriction (CR), but also the restriction of specific components of the diet, has been known for decades to affect longevity. However, the understanding of how each component of the macronutrients affects longevity and stress resistance is poorly understood, in part because of the complexity of many of the model organisms studied. Here we studied how each amino acid and glucose cooperate to activate cell sensitizing pathways and promote aging. We identified specific amino acids in the diet that affect cellular protection and aging, describe how different pathways mediate these pro-aging effects, describe the effect of glucose and specific amino acids on the levels/activity of stress resistance kinases and transcription factors, and identify specific nutrient depletions capable of increasing longevity and stress resistance. Because of the conserved pro-aging role of orthologs of many of the genes in the signaling network described in this paper, these results are likely to serve as a foundation for the elucidation of similar nutrient-dependent pro-aging mechanisms in mammals.

## Introduction

Calorie restriction (CR), which usually refers to a 20–40% reduction in calorie intake, can effectively prolong life span in taxonomically diverse organisms ranging from yeasts to mammals [1]. It is also known that selective restriction of carbohydrates or proteins, as well as alternate day fasting without an overall restriction of calories, can also extend longevity [2]–[3], suggesting that reduced levels of specific macronutrients in the diet (Dietary Restriction, which also includes CR), can achieve at least some of the effects of CR [3]. On the other hand, studies on model organisms focusing on genes and pathways involved in aging have identified Ras, the yeast ortholog of S6 kinase, Sch9 and the target of Rapamycin (TOR) as key regulators of longevity and stress resistance [4]–[5]. However, although the stress resistance genes as well as the transcription factors Msn2, Msn4, and Gis1 play key roles in the effects of CR on life span extension [6], the connection between the availability of each component of the diet, stress resistance and aging genes has remained only partially understood [7]. Depletion of glucose, the best characterized nutrient, causes calorie restriction-associated phenotypes including life span extension [8]–[9] while its addition to starved yeast cells alters the expression of almost one third of the yeast transcriptome [10]–[11]. These effects are largely due to altered activity of the protein kinase A (PKA) through Ras-cAMP and Sch9-dependent pathways [12]–[13]. Regarding the other macronutrients, many genes have been identified for their role as sensors and transporters for nutrients different from glucose [14]–[19], but little is known about the molecular cascades activated by specific nutrients. In yeast, amino acid scarcity increases replicative life span [20] possibly by affecting protein synthesis [21]. In flies and rodents, changes in amino acid or dietary composition can have profound effects on life span [22]–[26] and in human cell cultures the availability of amino acids affects gene expression profiles [27]–[29], but the effect of each amino acid on aging is largely unknown.

Evidence based on the effect of amino acid withdrawal and repletion points to the TORC1 complex as a major amino acids transducer in mammalian cells [30]–[31]. In CHO-IR mammalian cells, amino acid withdrawal results in the selective inhibition of S6K1 and dephosphorylation of 4E-BP, rendering these targets unresponsive to insulin [32]. On the contrary, amino acids replenishment or just the addition of leucine and arginine, in the absence of serum or insulin, restores 4E-BP phosphorylation, S6K1 activity and insulin sensitivity [32].

The AGC kinase Sch9 (ortholog of the mammalian S6 and possibly AKT kinases) has been reported to be a major target of the activated TORC1 complex [33]. The latter phosphorylates Sch9 at multiple residues within the hydrophobic motif (HM) whereas the Sch9 T loop is the target of the Phosphoinositide Dependent protein Kinase 1 (PDK1) orthologs Pkh1–3 [34]–[35]: the main downstream effectors of the PI3kinase that in yeast are activated by sphingolipids [36]–[37]. It has therefore been suggested that Sch9 integrates nutrient signals coming from Tor1 with stress signals coming from sphingolipids [35], [38]. Interestingly, a physical interaction between the general amino acid permease (Gap1) and three components (Pis1, Lip1, Tsc13) of the sphingolipids biosynthetic pathway has recently been demonstrated [39] raising the possibility that sphingolipids metabolism may be affected by amino acids availability. However, increasing evidences suggest that few molecular switches may integrate all nutrient signals. Flo11, one of the genes activated by the lack of nitrogen [40], is also affected by PKA activation state [41] integrating nitrogen signaling and glucose signaling. Other reports have related the activity of the General Amino-acids Permease (GAP1) to the PKA activation state [42]–[43] and even though they reached conflicting conclusions, both suggest a dependency of the amino acid transport system on the PKA activation state, thus connecting amino acid to glucose response. Pkh1–3 phosphorylate both PKA catalytic subunit and Sch9 T loop, linking sphingolipids to glucose signaling [34]. Moreover, Bcy1, the regulatory subunit of the PKA, is modulated by cAMP concentration, which transduces glucose availability, but is also phosphorylated by TORC1 [44].

Here, we investigated the effect of media composition on aging and stress resistance and identified the connection between specific nutrient and the well-known and conserved pro- and anti-aging genes.

## Results

### Dextrose and amino acids act on separate pathways

Although nutrient-dependent pathways in *Saccharomyces cerevisiae* have been studied extensively [21], [45]–[48], the role of each nutrient on cellular protection and aging is poorly understood. Because our previous studies and those of many other laboratories indicated that resistance to oxidative stress is tightly linked to longevity, we developed a protocol to measure oxidative stress resistance variations in post-diauxic yeast cells exposed to various nutrient mixtures (nutrient response assay, figure S1, see materials and methods section for details).

Experiments were performed using the DBY746 laboratory wild type yeast strain (carrying the Leucine, Histidine, Tryptophan and Uracil auxotrophies; see table S1 for the list of strains used and for the complete relevant genotype) and repeated with the corresponding isogenic prototrophic yeast strain, as well as with a natural yeast strain commonly used by winemaking industries.

**Figure 1:**
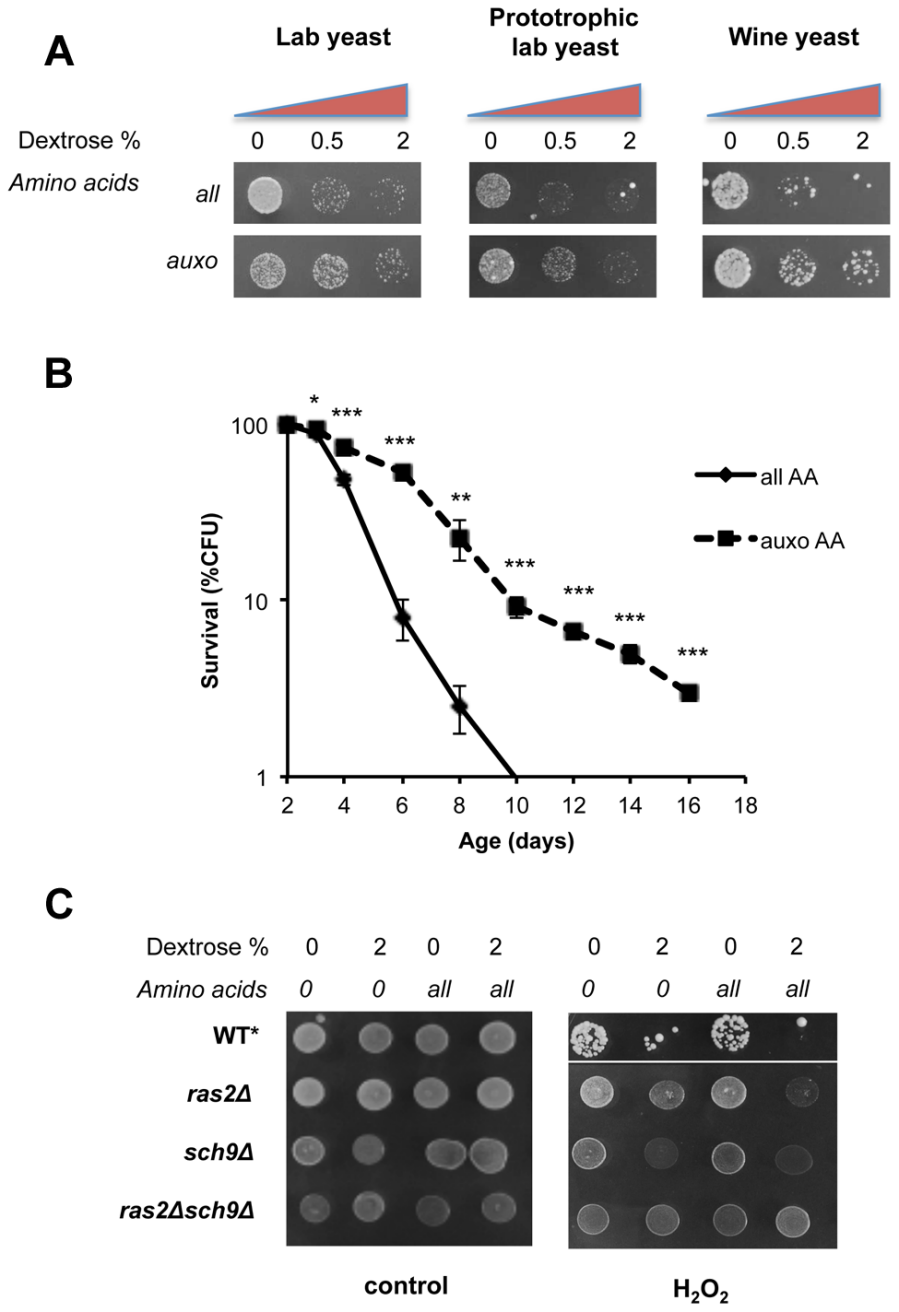
Dextrose and amino acids act on separate pathways. (A) Stress resistance (hydrogen peroxide treatment) of stationary phase laboratory and prototrophic wild type DBY746 yeast strain and winemaking yeast after short time nutrient replenishment (nutrient response assay, see figure S1 for a complete experimental scheme and materials and methods section for details). All mixtures added contained the basic SD components (Yeast nitrogen base, ammonium sulfate and phosphate). “Auxo AA” refers to a nutrient mixture containing the organic compounds required to compensate the wild type DBY746 auxothrophies (histidine, leucine and tryptophan and the nucleotide uracil) while all the other amino acids are missing. “all AA” refers to the complete SDC medium therefore containing all the amino acids (for a complete description see table S2). Dextrose concentration was added as specified. (B) *In situ* chronological life span (58) of wild type DBY746 in the presence of all or only the auxotrophic (auxo) amino acids (see above and table S2). The data represent the mean ± standard error of five different experiments. P values were evaluated by 2-tailed T-test for groups with unequal variants. *, p = 0.2; **, p<0.05; ***, p<0.005. (C) Stress resistance (hydrogen peroxide treatment) after the addition of the indicated different nutrients mixtures (nutrient response assay, see figure S1 for a complete experimental scheme) using the indicated isogenic derivatives of the DBY746 yeast strain.* The wild type strain was grown for three days, all the other strains were grown for 1 day only.

In agreement with previous observations [49]–[50], we found increased stress resistance by reduction of dextrose concentration from 2% to 0.5% both in auxotrophic and in prototrophic DBY746 and in winemaking yeast (Figure 1a and S2b, S3a) and synergistic effects of carbohydrates and all amino acids addition on cellular sensitization to either peroxide treatment or heat shock (Figure 1a S2a, b and S3a). To rule out the possibility that the effect of amino acids on stress resistance was merely due to pH changes, experiments were repeated at pH 3.7 and 6. Essentially the same results were obtained (Figure S2b, c). In addition, all the experiments were performed in non-limiting conditions for nitrogen source to separate the effects of limited nitrogen source from that of amino acid restriction.

To avoid the possibility that liquid cultures, such as those used in standard chronological life span assay, may lead to toxic levels of certain metabolites (e.g. acetic acid and ethanol [51]–[52]) or that regrowth phenotype may affect the interpretation of the results, we repeated the experiments using *in situ* chronological life span, in which about 200 cells are maintained isolated from others on a plate containing 2% glucose [53]. This assay confirmed the strong association between stress resistance and life span measurements (Figure 1b). In fact, consistently with the stress resistance assay, the presence of the complete amino acids mixtures significantly shortened the life span of wild type yeasts with respect to a medium containing only the essential amino acids.

To identify the downstream effectors of glucose- and amino acids-dependent sensitization, we monitored stress resistance in isogenic yeast strains lacking key mediators of nutrients signaling: (the *RAS2* and/or *SCH9* genes) in the presence of different nutrient mixtures (Figure 1c). Both gene products are known to modulate PKA activity, a known stress response inhibitor, by different means. Ras2 is essential for glucose-dependent PKA activation in nutrient starved yeast cells (Figure 1c), while Sch9 phosphorylates the PKA regulatory subunit in response to Tor1 activation [44]. Notably, the latter has been implicated in dextrose, nitrogen and amino acid metabolism in different organisms [54]. Our results (Figure 1c) confirm previous observations [17] on the ability of amino acids to increase stress sensitivity in glucose-derepressed cells. In addition, we confirmed the major role of Ras2 in glucose response and the role of Sch9 as a major amino-acid response transducer. It must be noted that pure amino acid treatment, even for prolonged time (24 h, data not shown), was completely ineffective in sensitizing cells to peroxide treatment in wild type, *ras2Δ, sch9Δ*or *ras2Δsch9Δ* genetic backgrounds (Figure 1c). These experiments confirmed previous results [15], but also provide new insights suggesting that amino acids and dextrose act on separate pathways and that glucose de-repression is necessary for amino acids-dependent cell sensitization.

### Tor1 and Pkh1/2 converge on Sch9

**Figure 2:**
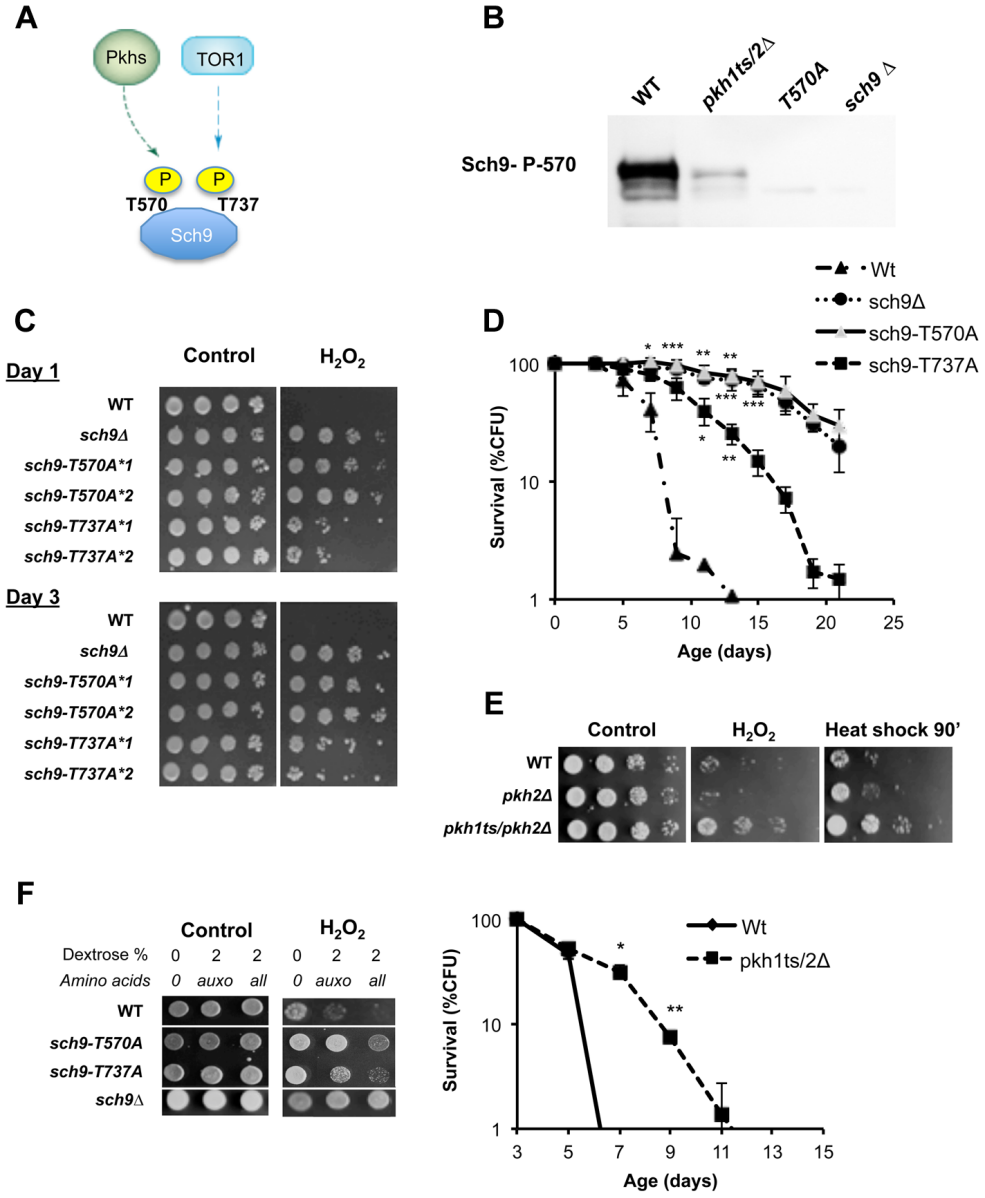
Role of Sch9 activation state in survival, stress resistance and amino acids response. (A) Activation scheme of Sch9 kinase. (B) Western blot of whole protein extract from the indicated W303 isogenic derivatives grown to exponential phase. T570A refers to protein extract from the strain carrying the *SCH9* allele coding for the amino acid substitution T570A. Anti-SCH9 P570 was used as a primary antibody. (C) Stress resistance (hydrogen peroxide treatment) of the indicated DBY746 isogenic derivatives grown for 24 h (Day1) or 72 h (Day3) in complete synthetic medium (SDC, all the amino acids supplied), the asterisks followed by a number refer to two different isolates with the same relevant genotype. (D) Chronological life span of the same group of strains. (E) Peroxide (H_2_O_2_) and heat shock (55°C) resistance of wild type DBY746 and of the isogenic indicated derivatives strains cultured to day 2 (upper panel). Chronological life spans of the same group of strains (lower panel). All the cultures, after two days of growth at 30°C, were transferred at 35°C to inactivate the Pkh1 thermo sensitive allele as previously described [86]. Experiments were performed in triplicate. Standard errors bars are shown. P values were evaluated by 2 tailed T-test for groups with unequal variants. *, p<0.02; **, p<0.005. (F) Nutrient response assay (hydrogen peroxide treatment after nutrient addition, see figure S1 for the experimental scheme) of strains (DBY746 derivatives) bearing different *SCH9* alleles. T570A and T737A refer to the amino acid substitutions coded by the mutated alleles.

Considering the central role of the AGC kinase Sch9 in amino acid response, stress resistance and aging, we studied its connection with upstream kinases in an attempt to identify the mechanisms linking specific nutrients to Sch9. AGC kinases are regulated by a general scheme mainly based on the phosphorylation of two amino acid residues which in yeast Sch9 are threonine 570 (located within the T loop) and threonine 737 (located within the hydrophobic motif, HM, Figure 2a). Phosphorylation of these residues is accomplished by specific kinases [55]–[59]. In yeast, the PDK1 orthologs Pkh1/2 phosphorylates the T-loop T570, while the TORC1 complex phosphorylates the HM T737 (Figure 2a) [60]–[61]. Western blots using an anti-P570 confirmed the disappearance of the Sch9 immuno-reactive band in protein extracts from strains with deficiencies in both Pkhs (Pkh1ts, pkh2Δ, see Figure 2b) (antibody specificity was confirmed using the T570A mutant (Figure 2b)), thus confirming previous observations obtained using the Pkhs inhibitor drug myriocin or mutants with impaired sphingolipid biosynthesis (Lcb1 mutants) [35]. Site directed mutagenesis, leading to T570A and T737A amino acid substitutions, which abolish T-loop and HM phosphorylation sites, respectively, resulted in increased stress resistance and longevity (Figure 2c, d). Surprisingly, the comparison of the relative effect of the two amino acidic substitutions T570A and T737A uncovered a more important role for the Pkh1/2 T-loop phosphorylation site in stress resistance and aging. T570A increased longevity and stress resistance to an extent similar to that caused by *SCH9* deletion (Figure 2c,d) whereas the T737A substitution only caused a partial increase in stress resistance and longevity. Cells, with impaired Pkh function, showed increased survival in both the DBY746 and the W303 genetic backgrounds (Figure 2e, S3b). Consistent with this result, resistance to multiple stresses, in both genetic backgrounds, increased when Pkh1/2 function was reduced (Figure 2e, S3c). These results confirmed the role of sphingolipid metabolism on stress resistance and longevity [35]. We then evaluated the stress resistance of DBY746 yeast cells bearing the allele coding for the sch9-T570A or the sch9-T737A in response to different nutrient mixtures (Figure 2f). The results point to a critical role for Sch9 as an integration point of Pkhs- and Tor-mediated amino acid responses (Figure 2f). In addition, the comparison of mutations within the T-loop and the HM domains points to the T-loop and its activators Pkh1–2 as a major pro-aging amino-acid response pathway.

### Specific amino acids decrease stress resistance by different pathways

**Figure 3:**
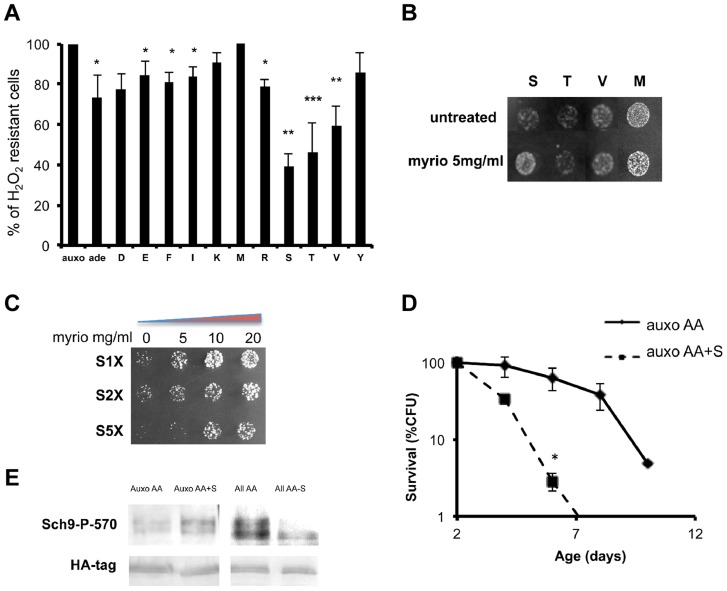
Role of single amino acids in stress sensitization. (A) Nutrient response assay (hydrogen peroxide treatment after nutrient addition) using cultures of stationary phase (two days of growth) wild type DBY746 strain (see figure S1 for the experimental scheme). Synthetic medium with dextrose and only the organic compounds necessary to compensate the auxothrophies (leucine, histidine, tryptophan and uracil) were added to ‘auxo’ sample. The other samples were added with the same mixture plus the single indicated non-essential amino acid. After hydrogen peroxide treatment, a small aliquot from each sample was plated on rich (YPD) medium and colony forming units (CFU) counted after 2 days. The CFU number obtained with the ‘auxo’ medium was the highest and was used as a reference (100%) for all the other samples. The latters were expressed as (CFU/CFU(auxo))*100. The data represent the mean ± standard errors of five independent experiments. Statistical significance was evaluated by 2-tailed T-test for groups with unequal variants. *, p<0.05; **, p<0.02; ***, p<0.001. Amino acids are indicated using the single letter code. (B) Nutrient response assay (hydrogen peroxide treatment after nutrient addition) using the wild type strain DBY746 (see figure S1 for the experimental scheme) combining the addition of the auxo mixture (see the legend to the figure 1A for details) plus the indicated amino acid in the presence/absence of the Pkhs inhibitor myriocin. (C) Dose-response nutrient response assay (hydrogen peroxide treatment after nutrient addition) using the wild type DBY746 strain (see figure S1 for the experimental scheme) obtained combining the addition of the auxo mixture (see A for details) plus different serine concentration with increasing amount of the Pkhs inhibitor myriocin. (D) Chronological life span of the DBY746 yeast strain grown with the auxo medium (see legend to figure 3A for a complete description of this medium) in the absence (auxo AA) or in the presence (auxo AA+S) of the standard concentration of the amino acid serine. P values were evaluated by 2 tailed T-test for groups with unequal variants. *, p<0.002. (E) Effect of serine presence on Sch9 threonine 570 phosphorylation. Upper panel: Western blot of whole protein extracts from wild type yeast stained with a specific anti-P570 antibody. Cells (exponential phase) were grown in the presence of the auxo mixture (Auxo AA) (see legend of figure 1A for a complete description), plus the standard concentration of serine (Auxo AA+S), with the complete amino acid mixture (All AA) and with the complete amino acid mixture lacking only the serine amino acid (All AA-S; standard concentration of amino acids are indicated in table S2). Lower panel: wild type (W303) strain, HA tagged on *SCH9* gene was treated in the same conditions as the upper panel and stained with an anti-HA primary antibody.

To understand the connection between specific amino acids and Sch9 activation by phosphorylation of the T-loop and HM domain, we measured stress resistance in wild type yeast cells treated with mixtures containing minimal medium plus one single non-essential amino acid. The result, shown in Figure 3a, identified the amino acids serine, threonine and valine as the most effective cell-sensitizing amino acids. Previous studies, based on survival in media lacking specific substances, identified glutamic acid as a pro-aging amino acid [38], [62]–[63] whereas methionine has been described as a pro-aging amino acid in other eukaryotic systems [23], [64]–[65].

We then tested the ability of combinations between the identified amino acids to increase stress sensitivity. The results confirmed the stress enhancing capability of serine, threonine and valine and to a lesser extent of glutamic acid and methionine (Figure S4a). Since Pkhs, the kinases known to phosphorylate the T-loop of the Sch9 protein, can be inhibited by the drug myriocin, which blocks sphingosine biosynthesis [66], we compared single amino acid-dependent stress sensitization in the presence/absence of myriocin. Surprisingly, the drug rescued serine sensitization but had no effect on methionine/threonine/valine treatment (Figure 3b). The involvement of the Pkh sphingolipid pathway in serine response was confirmed by a dose-response assay with increasing myriocin concentration at various serine levels (Figure 3c). The results confirmed the myriocin-dependent rescue of the stress resistance, even at very high serine concentrations (5×). The dependency of serine sensitization on Pkhs function was further confirmed by assessing modulation of serine sensitization in the presence/absence of functional Pkh alleles (Figure S4b). In addition, overexpression of the human ortholog of the Pkhs kinases, PDK1, increased serine sensitivity suggesting the existence of a conserved role of PDK orthologs in lower and higher eukaryotes (Figure S4c). Chronological life span assay confirmed that serine is a pro-aging amino acid since serine supplementation, at the standard concentration, significantly shortened the life span of wild type yeast strains (Figure 3d). Finally, western blot analysis using the anti phosphothreonine 570 Sch9 specific antibody, confirmed that serine addition was capable of increasing the phosphorylated moiety of the threonine 570 amino acid residue (Figure 3e). In addition, removal of serine from the amino acid mixture was sufficient to significantly decrease the P570 level, pointing to serine as the major amino acid regulator of Sch9T570 phosphorylation (Figure 3e). The possibility that the amino acid administration influences the amount of Sch9 protein rather than its phosphorylation status was ruled out by tagging the Sch9 protein with the hemoagglutinin epitope and using a commercial anti-HA antibody to monitor the amount of the Sch9 protein in the various conditions (Figure 3e).

### Threonine and valine activate Tor/S6K-dependent pro-aging signaling

**Figure 4:**
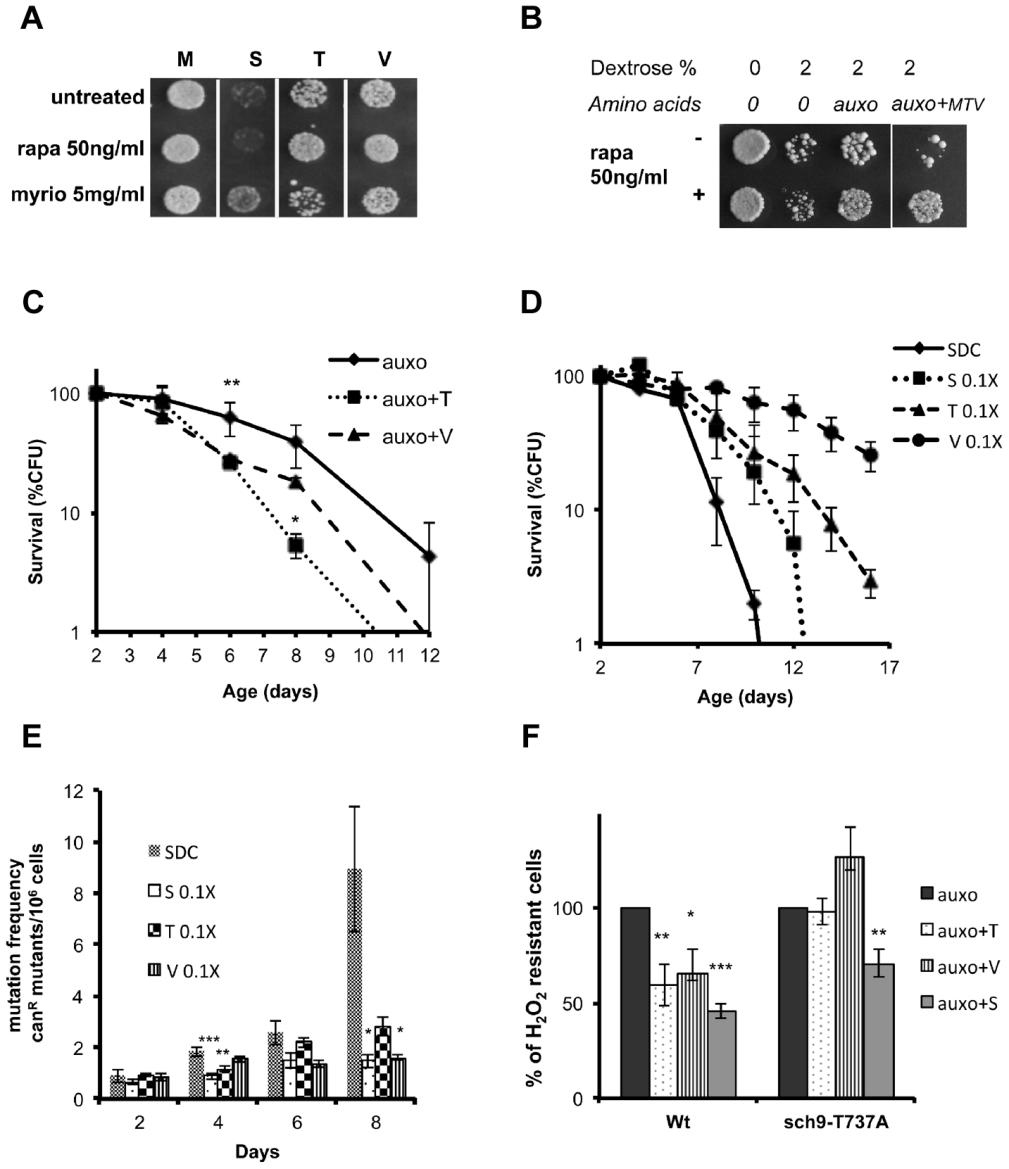
Role of Tor/S6K in amino acid response. (A) Nutrient response assay (hydrogen peroxide treatment after nutrient addition) using the wild type DBY746 strain (see figure S1 for the experimental scheme) combining the addition of the auxo mixture (see the legend to the figure 1A for details) plus the indicated amino acid with the presence/absence of the Tor-inhibiting drug rapamycin or with the Pkhs-inhibiting drug myriocin. (B) Nutrient response assay (hydrogen peroxide treatment after nutrient addition) of the DBY746 yeast strain with/out rapamycin after the addition of the indicated mixture of nutrients (see the legend to the figure 1A for details). The components of the synthetic medium were added to all samples. 2% Dextrose was added were indicated. Auxo refers to the addition of all the organic compounds necessary to compensate the DBY746 auxothrophies (leucine, histidine, tryptophan and uracil) while auxo+MTV also contained the amino acids methionine, threonine and valine. (C) Viability of the wild type DBY746 yeast strain grown in the presence of the auxo mixture (auxo, see the legend to the figure 1A for a complete description of this medium) or in the presence of auxo mixture plus the amino acid threonine or valine (auxo+T, auxo+V respectively). Experiments were done in triplicate and mean values are represented with standard errors. P values were evaluated by 2 tailed T-test for groups with unequal variants. *, p<0.1; **, p<0.005. (D and E) Chronological life span and mutation frequency of DBY746 wild type strain, grown in complete medium (SDC) or in SDC with reduced concentration of serine, threonine or valine (0.1×, 1∶10 of the standard concentration, see table S2 for a definition of the standard concentration used). Experiments were done in triplicate and mean values are represented with standard errors. P values were evaluated by 2 tailed T-test for groups with unequal variants. *, p<0.1; **, p<0.04; ***, p<0.02. (F) Nutrient response assay (hydrogen peroxide treatment after nutrient addition) using cultures of stationary phase (two days of growth) wild type DBY746 strain and of the isogenic derivative carrying the Sch9 allele coding for the amino acid substitution T737A (see figure S1 for the experimental scheme). Synthetic medium with dextrose and only the organic compounds necessary to compensate the auxothrophies (leucine, histidine, tryptophan and uracil) were added to ‘auxo’ sample. The other samples were added with the same mixture plus the single indicated non-essential amino acid. After hydrogen peroxide treatment, a small aliquot from each sample was plated on rich (YPD) medium and colony forming units (CFU) counted after 2 days. The CFU number obtained with the ‘auxo’ medium was used as a reference (100%) for all the other samples. The latters were expressed as (CFU/CFU(auxo))*100. *, p<0.1; **, p<0.02; ***, p<0.001 comparing each value against the corresponding (strain specific) “auxo” value.

To understand whether Tor1 may serve as the link between the other pro-aging amino acids, Sch9 activation and cellular sensitization, we examined the effect of the three amino acids, identified as the most effective in stress sensitization (Figure 3a), in the presence or absence of the TORC1 drug inhibitor rapamycin. Rapamycin was capable of suppressing the sensitization caused by threonine and valine, but was completely ineffective in suppressing the serine-dependent effects (Figure 4a) in a pH-independent manner (Figure S5a, b). In addition, treatment with different nutrient mixtures, in the presence/absence of rapamycin, suggested that TORC1 has a negligible role in dextrose–dependent sensitization and confirmed its capability in suppressing threonine and valine (MTV)-dependent sensitization (Figure 4b). Chronological lifespan, obtained in the presence of minimal medium supplemented with either threonine or valine at the standard concentration, confirmed the pro-aging role of these amino acids (Figure 4c). On the other hand, incubation of yeast with standard synthetic medium, restricted (1∶10 of the standard concentration) for only one of the identified pro-aging amino acids, was sufficient to increase the life span and to reduce the rate of spontaneous point mutations (Figure 4 d, e). Finally, the insertion of the point mutation T737A within the hydrophobic motif of the Sch9 protein kinase was capable to abolish the sensitization to oxidative stress due to threonine or valine administration while was ineffective against serine administration (Figure 4f). These results reveal the connection between specific amino acids and two different amino acid-dependent pro-aging pathways: the Tor1-dependent one and the newly discovered Pkhs-dependent one [35], both converging on Sch9 but on two different phosphorylation sites. Notably, we also demonstrate that restriction of specific non-essential amino acids increases life span and decreases the mutation rate.

### Specific amino acids cause Rim15 repression

**Figure 5:**
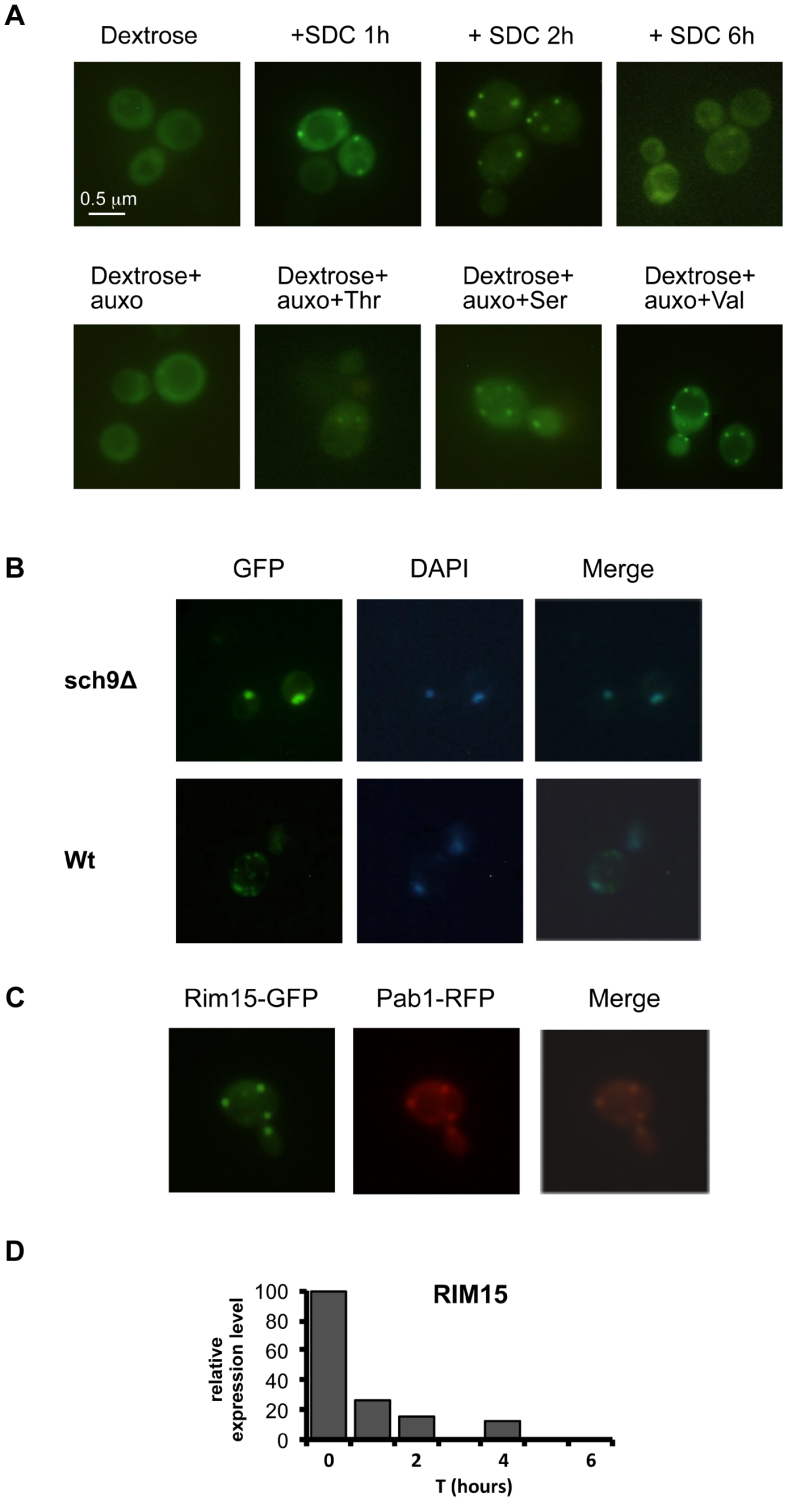
Amino acids affect Rim15 activity. (A) Wild type DBY746, was transformed with a plasmid coding for the Rim15-GFP fusion protein, after two days of growth the exhausted medium was replaced (upper panel) with fresh synthetic medium lacking all the amino acids (dextrose) or with the complete medium (SDC), pictures were taken after the indicated time. In the lower panel the fresh medium contained also compounds necessary to compensate the auxothrophies (the amino acids L, H, W and the nucleotide Uracil) plus the indicated amino acid (pictures were taken after two hours of incubation). (B) Wild type (Wt) and *sch9Δ* mutants (*sch9Δ*) were transformed with a plasmid coding for the Rim15-GFP fusion protein. After two days of growth wild type cells were transferred onto fresh medium while sch9Δ cells were left in the exhausted medium. GFP fluorescence or DAPI staining were observed after 2 additional hours in the aforementioned medium. (C) Wild type DBY746 strain, transformed with Rim15-GFP and Pab1-RFP expressing plasmids was grown to the post-diauxic phase (two days of growth) and then transferred onto fresh complete SDC medium, fluorescence was observed after two hours of incubation. (D) Rim15 mRNA level fluctuation in response to nutrients addition. mRNA obtained from stationary phase DBY746 cell culture (T = 0) or from the same culture but collected after 1, 2 or 4 hours after the switch to fresh SDC was subjected to Real Time PCR with Rim15 specific oligonucleotides. The amount of mRNA used for each PCR was normalized using the actin (ACT1) transcript levels as an internal control. The expression level of Rim15 before the medium switch (T = 0), which was the highest, was used as a reference point (100% of expression). The expression at the others time points was calculated using the formula [expr level(T = 1, 2, 4)/expr level (T = 0)] * 100.

**Figure 6:**
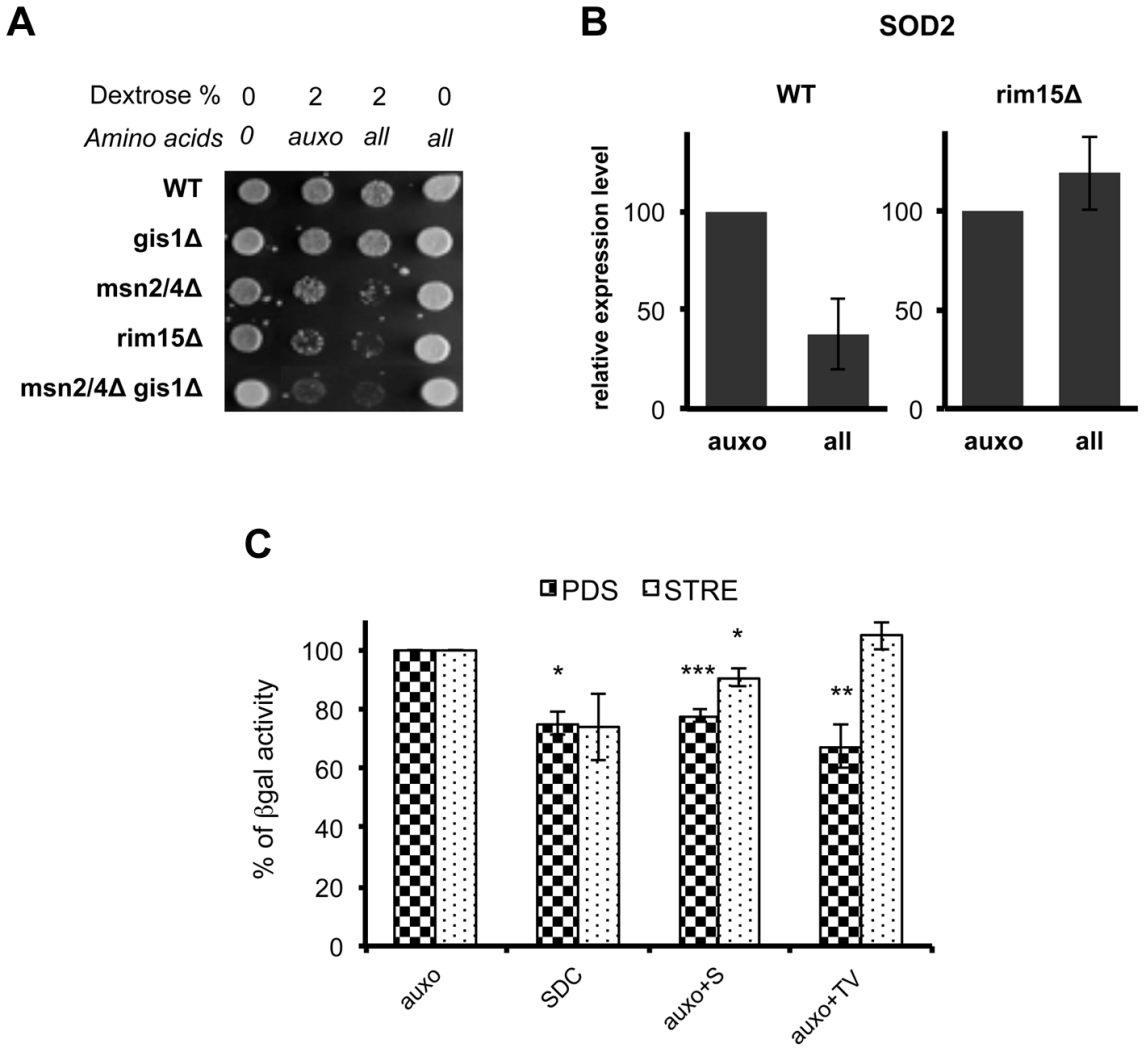
Amino acids affect stress response genes in a Rim15-dependent way. (A) Nutrient response assay of DBY746 (WT) and of the isogenic derivatives involved in stress response gene transcription. Auxo means the addition of the organic compounds necessary to compensate auxotrophies. (B) DBY746 bearing a wild type (WT) or a null (rim15Δ) *RIM15* allele were grown until day 3, then switched into fresh synthetic medium containing only ‘auxo’ amino acids or ‘all’ amino acids (for a detailed description of auxo mixture see the legend to the figure 1A); after 4 hours RNA was extracted and retro transcribed as reported in materials and methods section; quantitative RT-PCR was performed using *SOD2* specific primers and gene expression levels were normalized using *ACT1* as an internal control. The bar chart shows *SOD2* expression level after SDC incubation with respect to *SOD2* expression after incubation only with L, H, W as amino acids source. The data represent the mean ± standard errors of three different experiments. (C) Beta galactosidase assay of PDS and STRE reporters. Post-diauxic yeast cell (about 10^8^ cells at day 2 in liquid culture) were exposed to nutrients (4 hrs with the indicated mixtures). Cells were then disrupted and beta galactosidase activity was measured. The activity obtained with medium containing only the amino acids necessary to compensate strain auxotrophies (auxo) was referred to as 100% activity. Statistical significance was evaluated by 2-tailed T-test for groups with unequal variants. Data shown are mean and standard deviation of three independent samples assayed. * , p<0.5; **, p<0.05; ***, p<0.01.

To understand the connection between specific amino acids and aging, we investigated the localization of the protein kinase Rim15 in response to the presence of the key pro-aging amino acids. Rim15 is a serine/threonine protein kinase whose function is central in G_0_ arrest [67] and in cellular aging [6], [68]. Tor1, Sch9 and PKA control its cellular localization and activity [69]. Observing the fluorescence obtained using a plasmid coding for Rim15-GFP fusion protein, we first confirmed the role of the Sch9 protein kinase in Rim15 cellular localization. In fact, while wild type cells localize Rim15 outside the nucleus in log phase cultures and after nutrients addition to stationary phase cultures, the lack of *SCH9* caused Rim15 nuclear localization in day 2 yeast cells (Figure 5b) and after nutrient re-feeding (Figure S6c). In addition, wild type post-diauxic yeast cells (day 2) were exposed to nutrient re-feeding and Rim15-GFP fluorescence was monitored overtime. We observed an initial re-localization (appearance of the granules) followed by a time-dependent decrease (2–6 hrs interval) of the Rim15-GFP fluorescence (Figure 5a upper panel) but only with wild type Sch9 protein (Figure S6c). To characterize the nature of the granules, appearing after nutrient re-feeding, we co-expressed, in wild type cells, the plasmid coding for Rim15-GFP fusion protein together with the plasmid coding for the Pab1-RFP fusion protein, the latter being a known marker for stress granules [70]. Fluorescence analysis, 2 hours after nutrient replenishment in day 2 cultures, showed co-localization of the two fusion proteins indicating Rim15 localizes within stress granules after nutrients supplementation (Figure 5c). To determine if the Rim15-GFP disappearance, observed after longer nutrient incubation, was due to increased protein degradation or silenced transcription, we performed quantitative PCR of Rim15 transcript on RNA extracts, obtained at different time points, after nutrient replenishment. The results confirmed the role of reduced RNA levels in Rim15 activity regulation (Figure 5d). We then monitored if the addition of the identified pro-aging amino acids was sufficient to obtain Rim15 cellular re-localization. Addition of single amino acids to the otherwise minimal medium revealed the central role of threonine, serine and valine in activating the Rim15 re-localization pathway (Figure 5a lower panel) but only in glucose de-repressed cells (not shown), thus suggesting that the Tor1 and Pkh pathways may directly or indirectly regulate Rim15 protein activity. On the other hand Rim15 is reported to activate stress resistance transcription factors Msn2/4 and Gis1. It is known that Msn2/4 transcription factors bind to the STRE (STress Responsive Elements) motif contained in the promoter of stress responsive genes [71]–[73] whereas Gis1 transcription factor binds to the PDS (Post Diauxic Shift) motif contained in the promoter of several genes activated at this metabolic transition [74]–[76]. We tested the effect of nutrients on isogenic yeast strain carrying single, double or triple deletions of these transcription factors (Figure 6a). Our experiments revealed a central role for Rim15 and Msn2/4 but a much more modest effect of the deletion of Gis1 transcription factors alone on the glucose- and amino acids-dependent stress sensitization (Figure 6a). However, the unexpected high stress resistance and STRE element activation in the *gis1Δ* strain is likely due to a compensatory Msn2/4 activation in the presence of a *gis1* null allele. In fact, the Msn2/4-dependent STRE/beta galactosidase activity was increased in the *gis1Δ* null cells (Figure S6a) but deletion of both Msn2/4 and Gis1 caused a sensitization similar to that observed in *rim15* deletion mutants (Figure 6a). Quantitative PCR of the prototype stress response gene *SOD2* confirmed the role of amino acid administration in controlling stress response gene expression but only in the presence of the Rim15 protein kinase (Figure 6b). Beta-galactosidase assay using PDS (Gis1-dependent) or STRE (Msn2/4) gene reporters in various nutrient conditions indicated the contribution of amino acids to inhibition of both STRE and PDS-dependent gene transcription (Figure 6c). The usage of single, as well as, mixtures of the most sensitizing amino acids confirmed the role of serine and threonine/valine dependent pathways in regulating mainly PDS-driven gene expression (Figure 6c).

## Discussion

The understanding of the mechanisms linking DR to its anti-aging effects in higher eukaryotes has been hindered by their complexity. *S. cerevisiae* provides a very simple organism in which the effect of each major pro-aging nutrient can be dissected. In the present work we describe the cooperation between glucose and the amino acids threonine, valine and serine, in sensitizing yeast cells to stress and promoting aging via two major pathways. These results enhance our understanding of previously poorly understood roles and interactions between specific nutrients to promote aging but also point to specific amino acids and their effect on different pathways previously established to cause aging. By contrast, the effect of methionine restriction in extending the life span of fruit flies [23] and rodents [77]–[80] does not appear to be as important for the protection of yeast cells. We also show a less potent than serine, valine and threonine but detectable role of glutamate in increasing stress sensitivity in agreement with data on the ability of its deficiency to extend yeast survival [62]–[63].

**Figure 7:**
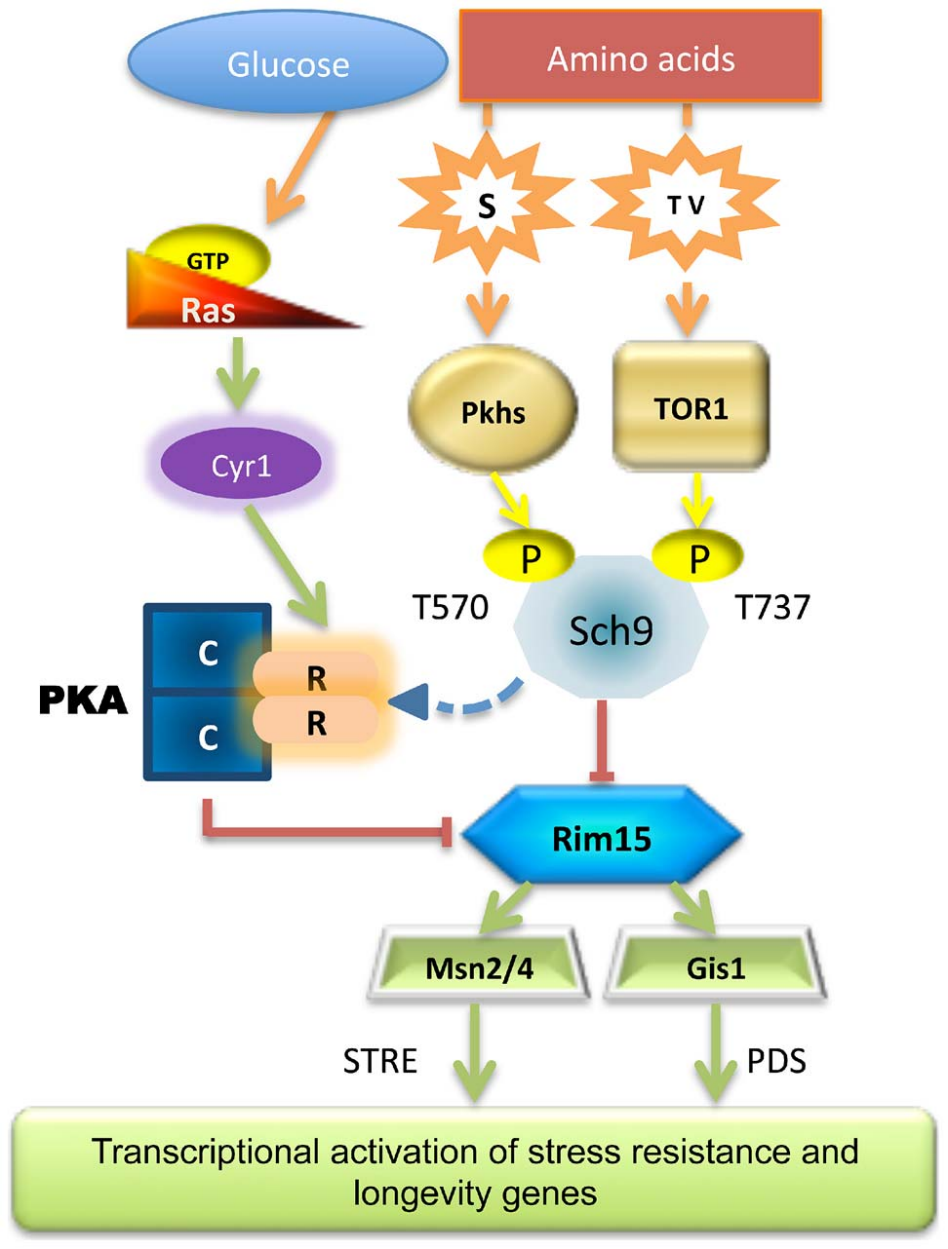
Scheme of the nutrient pathways. Schematic diagram of how specific nutrient activates the known pro-aging pathways, the central role of Sch9 and Rim15 is shown.

Our genetic and biochemical analysis revealed that the yeast amino acid response relies on at least two different pathways: the well-characterized TORC1-S6K pathway, which has been described as an integrator of different nutrient and energy signals in organisms including humans [81]–[82] and the sphingolipid-dependent Pkh1/2 pathway, also shown to promote aging in yeast (Figure 7) [35]. Threonine and valine activated the TORC1 pathway and promoted cellular sensitization that could be reversed by the well-established anti-aging drug rapamycin, whereas serine specifically activated Pkhs and promoted cellular sensitization by a mechanism, which could be reversed by the Pkh inhibitor myriocin. Considering that L-serine is the substrate of the serine palmitoyltransferase, the α-oxamine synthase enzyme that catalyzes the condensation reaction of L-serine and palmitoyl-CoA to form 3-ketodihydrosphingosine, we propose that serine may activate Pkh by enhancing the first and rate-determining step of the sphingolipid biosynthesis pathway [83]. Thus, serine administration may be equivalent to enhance sphingolipid biosynthesis, which is known to activate Pkhs and promote aging [35]. This hypothesis is supported by the overlap between the treatment with the sphingolipid biosynthesis inhibitor myriocin and Pkhs impairment.

Our analyses demonstrate that Sch9 plays a critical role in these nutrient response mechanisms since it integrates signals from the two pathways adapting its phosphorylation status and activity accordingly. This may explain the dominant role of the Tor/Sch9 pathway in promoting aging in yeast and possibly in higher eukaryotes. Rim15 subcellular localization also primarily relied on specific amino acid availability (threonine, serine and valine). Thus, although activated Rim15 is clearly central in nutrient-dependent cellular protection, its regulation by amino acids appears to be complex and to depend on both the Pkh and Tor/S6K pathways. In agreement with our hypothesis partial depletion of each of the amino acids described in this work was capable of increasing the life span. As expected from our previous studies, transcription factors Msn2/4 and Gis1 are downstream mediators of the anti-aging effects of Rim15 (Figure 6).

In summary, these results shed new light on a nutrient response network in which different genes are linked to specific components of the diet. Because orthologs of many genes in this network are known to affect aging in higher eukaryotes, these results are likely to point to similar mechanisms in mammalian cells.

## Materials and Methods

### Strains, media and plasmids

Strains and plasmids used in this study are listed in table S1. Gene Knockouts were generated by one-step gene disruption [84]. The sch9T570A and sch9T737A mutants were constructed using plasmids PFR82 and pAM202 (kindly provided by Dr. Thorner, University of California, Berkeley [57]) by homologous integration at the SCH9 locus.

Cells were grown in YPD (1% yeast extract, 2% peptone, 2% glucose), minimal medium SDC (0.17% yeast nitrogen base, 0.5% ammonium sulfate, 0.08% amino acids, pH 6) or selective media, with appropriate amino acids content (see table S2 for a complete list) to maintain selection for plasmids, containing 2% glucose as carbon source. Cells were grown at 30°C.

### Chronological life span and mutation frequency assay

Overnight SDC cultures were diluted (1∶10) into flasks covered with aluminum foil caps with fresh SDC medium to a final volume of 10 ml (with a flask to culture ratio of 5∶1) and kept at 30 °C with shaking (200 rpm) to ensure proper aeration. This dilution time point was considered day 0. Every other day, aliquots from the culture were properly diluted and plated onto YPD plates. The YPD plates were incubated at 30 °C for 2–3 days. Viability was assessed by Colony Forming Unit (CFUs) count. The CFUs obtained at day 2 were considered to be the initial survival (100%).

In situ viability was done as previously reported [53]. Briefly, aliquots of a two days old liquid culture of a trp- strain are plated on many plates of synthetic medium containing the indicated mixture of amino acids but lacking tryptophan. Plates are incubated at 30 °C and no growth is observed due to the lack of tryptophan. Every two days, plates are supplemented with the appropriate amount of tryptophan and put back in the incubator where cells start now dividing. Colony forming units are registered after two more days of incubation and scored as the percentage of CFU with respect to CFU at day 2, the latter considered as 100% of survival.

Mutation frequency was evaluated, as previously described [53], by monitoring the percentage of cells that become resistant to canavanine treatment during chronological life span experiment.

### Stress resistance assay

Heat shock resistance was measured by spotting serial dilutions of cells removed from SDC cultures onto YPD plates and incubating at either 55 °C (heat-shocked) or 30 °C (control) for 60 minutes to 120 minutes. After the heat-shock, plates were transferred to 30 °C and incubated for 2–3 days.

For oxidative stress resistance assays, aliquots of cells were diluted in K-phosphate buffer 0.1M, pH 6, and treated with different concentrations of hydrogen peroxide for 30 minutes. Serially diluted cells were then spotted onto YPD plates and incubated at 30 °C for 2–3 days.

### Nutrient response assay

Overnight SDC cultures were diluted 1∶10 into fresh SDC medium and were maintained at 30°C with shaking until day 2 or 3 of growth, then cultures were disposed into 24-multiwell plates and centrifuged at 3500 rcf for 20 minutes, pellets were re-suspended into different fresh media, that contained different mix of amino acids and/or different glucose concentrations, cells were incubated at 30°C shaking for 4 hours (amino acids used for the mixes and their concentrations are listed in table S2). After incubation cells were pelleted and re-suspended in K-phosphate buffer 0.1M, pH 6 and treated with hydrogen peroxide for 30 minutes. Cells from each well were then spotted onto YPD plates and incubated at 30 °C for 2–3 days (for a scheme of the nutrient response assay see fig. S1).

### Protein isolation

Protein extract were prepared by glass-bead disruption in a protein extraction buffer (50 mM MES KOH pH 6.2, 0.05 mM EDTA, 0.1 mM MgCl_2_, 0.5 mM DTT, 1× Protease inhibitor mix (Sigma), 1 mM PMSF, 25 mM NaF, 10 mM NaN_3_, 10 mM sodium beta-glycero-phosphate, 10 mM Na_2_H_2_P_2_O_7_). The lysate was spun down at 5000 rpm for 15 minutes and the supernatant tested. Protein concentrations were determined using Bradford Assay.

### Immunological analysis

Proteins were separated by SDS-PAGE [85]. Resolved proteins on gels were transferred onto nitrocellulose membrane (Schleicher & Schuell) using 192 mM glycine, 25 mM Tris, 20% methanol in a Mini Trans-Blot Electrophoretic Transfer Cell (Biorad). Blots were then blocked with 1% bovine serum albumin (BSA) in 20 mM Tris, 0.5M NaCl, pH 7.5 (TBS), washed in TBS with the addition of 0.05% Tween-20 (TBST) and incubated over night with primary antibody. Membranes were washed in TBST and incubated for 2 hours with secondary antibody (AP or HRP conjugated), then washed in TBS and labeled with BCIP/NTB or chemiluminescent ECL liquid substrate system (Promega and Invitrogen respectively).

The primary antibodies used were: anti-BCY1 (goat polyclonal, Bcy1 [yN19] sc-6765, Santa Cruz Biotechnology), anti-HA (mouse monoclonal, HA probe sc-7392 Santa Cruz Biotechnology), anti-Sch9P570 (a kind gift of Robby Loewith).

### GFP and RFP microscopies

Cells expressing Rim15-green and/or Pab1-red fluorescent fusion proteins were grown to stationary phase, treated with different nutrients mixtures for various times, then cells were used for fluorescence microscopy directly without fixation.

Nuclei were stained with 0.5 ug/ml of Hoechst 33342 (Invitrogen) for 15 minutes before cells were watched. Cells were viewed with an Olympus BX50 fluorescence microscope using the appropriate filters.

### RNA extraction and quantitative PCR assay

Total RNA was isolated using RiboPure- Yeast kit (Ambion) according to the kit's instructions. RNA was treated with RNase-free DNase I (Promega) to remove contamination of genomic DNA. 0.5 µg of total RNA was reverse transcribed into cDNA using ImProm-II Reverse Transcriptase (Promega) with sequence specific primers (ACT1, GAATCCAAAACAATACCAGTAG; SOD2, AGCTGCTAATTTAACCAAGAAG; RIM15, TTATCGTACTTTCATCGTCAC). Quantitative PCR experiments were performed on StepOne Real-Time PCR instrument (Applied Biosystems) using Fast SYBR Green Master Mix (Applied Biosystems) and the gene specific primers: ACT1, fw-TCGTGCTGTCTTCCCATCTATC and rev-GTAGAAGGTATGATGCCAGATC; SOD2, fw-CTCCGGTCAAATCAACGAAT and rev-CCTTGGCCAGAAGATCTGAG; RIM15, fw-GGAGCTGGAACTGGACGGCAAG and rev-AGCATGTCTGTGGCCTTTTGAA. Thermo-cycling conditions were as follows: 95 °C for 20 seconds followed by 40 cycles of 95 °C for 3 seconds and 60 °C for 30 seconds. Relative gene expression was calculated using the 2^−ΔΔCT^ method and normalized to ACT1 mRNA levels.

### Beta galactosidase assay

Cell pellet from 1 ml of culture was lysed with low salt buffer (50 mM Tris pH 7.5, 0.1× protease inhibitor cocktail (Sigma), 100 mM NaCl, 2 mM EDTA, 2 mM EGTA, 50 mM NaF). The protein concentration of the lysate was determined by Bradford assay. 55 µl of appropriately diluted samples of lysate was mixed with 85 µl of substrate solution (1.1 mg/ml ONPG in 60 mM Na_2_HPO_4_, 40 mMNaH_2_PO_4_, 10 mM KCl, 1 mM MgSO_4_, 50 mM 2-mercaptoethanol, pH 7.0). Absorbance at 420 nm was read every 5 minutes until 30 minutes after the initiation of reaction. Percentage of activity at every condition was determined respect of a control condition fixed as 100% of activity. Statistical significance was evaluated by 2 tailed T-test for groups with unequal variants, control versus individually all the others conditions.

## Supporting Information

Figure S1 Nutrient response assay. Flowchart of the protocol used to assess the effect of single as well as mixture of nutrients addition on stress resistance at stationary phase.
(TIF)

Figure S2 Acidification doesn't affect amino acid sensitivity. (A) Heat shock resistance of DBY746 wild type yeast strain grown to day 2 after a 4 hour pulse with a media containing 2% dextrose, all nitrogen source and either the amino acids necessary to compensate DBY746 auxotrophies (*auxo*) or the media with the complete mixture of amino acids (*all*). (B) Nutrient response assay in the presence of either the amino acids necessary to compensate DBY 746 auxotrophies (*auxo*) or the complete mixture of amino acids (*all*) at different percentages of dextrose at pH 6 and 3.7. (C) Nutrient response assay of prototrophic DBY746 yeast strain at pH 6 and 3.7 with different nutrient mixtures. Auxo indicates the addition of the compounds necessary to compensate the auxothrophies, all indicate the addition of the complete amino acid mixtures and auxo+S refers to the addition of the auxo mixture plus the amino acid serine at the standard concentration (for the list of the amino acid concentration used see table S2).
(TIFF)

Figure S3 Dextrose concentration affects stress sensitivity in cooperation with amino acids in a Ras2-dependent fashion. (A) Nutrient response assay of two commonly used yeast wild type strains (w303-1A and DBY746) with increasing dextrose concentrations with the compounds necessary to compensate auxotrophies (*auxo*) or with the complete mixture of amino acids (*all*). Viability (B) of wild type W303 and of the indicated isogenic derivative. Viability from day 2 was switched to 35 C to inactivate the Pkh1-thermo sensitive allele. Stress (H_2_O_2_) and heat shock (55°C) resistance (C) of wild type W303 and *pkh1ts/pkh2Δ* isogenic derivative strains.
(TIF)

Figure S4 (A) Effect of amino acid mixtures on nutrient response. Amino acids are indicated with the single letter code. (B) Nutrient response assay of yeast cells expressing a thermo sensitive Pkh1 allele and a null Pkh2 allele and the corresponding strain co-transformed with Pkh1 and Pkh2 overexpression plasmids (PKH1/PKH2). (C) Nutrient response assay of DBY746 strain (wt), with the overexpression of mammalian ortholog of Pkhs (PDK1) or with the overexpression of Sch9 (SCH9).
(TIF)

Figure S5 Rapamycin and myriocin treatments affect different amino acid signals. (A) Nutrient response assay in the presence of the Tor inhibitor Rapamicin (Rapa) at two different concentrations or with the same concentration of the solvent used to solubilize Rapamycin (DMSO). (B) Rapamycin versus Myriocin (Mirio) effect on nutrient response.
(TIFF)

Figure S6 (A) STRE LacZ activity of the indicated isogenic strains. Experiments were performed in triplicate. Standard errors bars are shown. P values were evaluated by 2 tailed T-test for groups with unequal variants. * p = 0.1; **p<0.01. (B) Western blot using a commercially available anti Bcy1 antibody. Whole extract of the indicated strains in log phase (DBY746 genetic background) were used. (C) Fluorescence of sch9D strain carrying the Rim15-GFP fusion protein. Cells were grown to stationary phase, the exhausted medium was changed with fresh SDC medium, the fluorescence was measured after the indicated time.
(TIFF)

Table S1 Yeast strains and plasmids used in this study. All the strains used in this study along with relevant genotype, reference and figure(s) where they appear are indicated.
(DOC)

Table S2 Composition of the medium used in all experiments except where otherwise specified. Synthetic complete glucose medium, SDC was modified with 4-fold excess of histidine, leucine, tryptophan, and uracil to compensate the auxotrophy of the DBY746 strain. pH was adjusted to 6.0 with NaOH.
(DOCX)
