# Supplementary figures and images for: Serine- and Threonine/Valine-Dependent Activation of PDK and Tor Orthologs Converge on Sch9 to Promote Aging
Source: PLoS Genet. 2014 Feb 6;10(2):e1004113. doi: 10.1371/journal.pgen.1004113 (PMC3916422; doi:10.1371/journal.pgen.1004113)

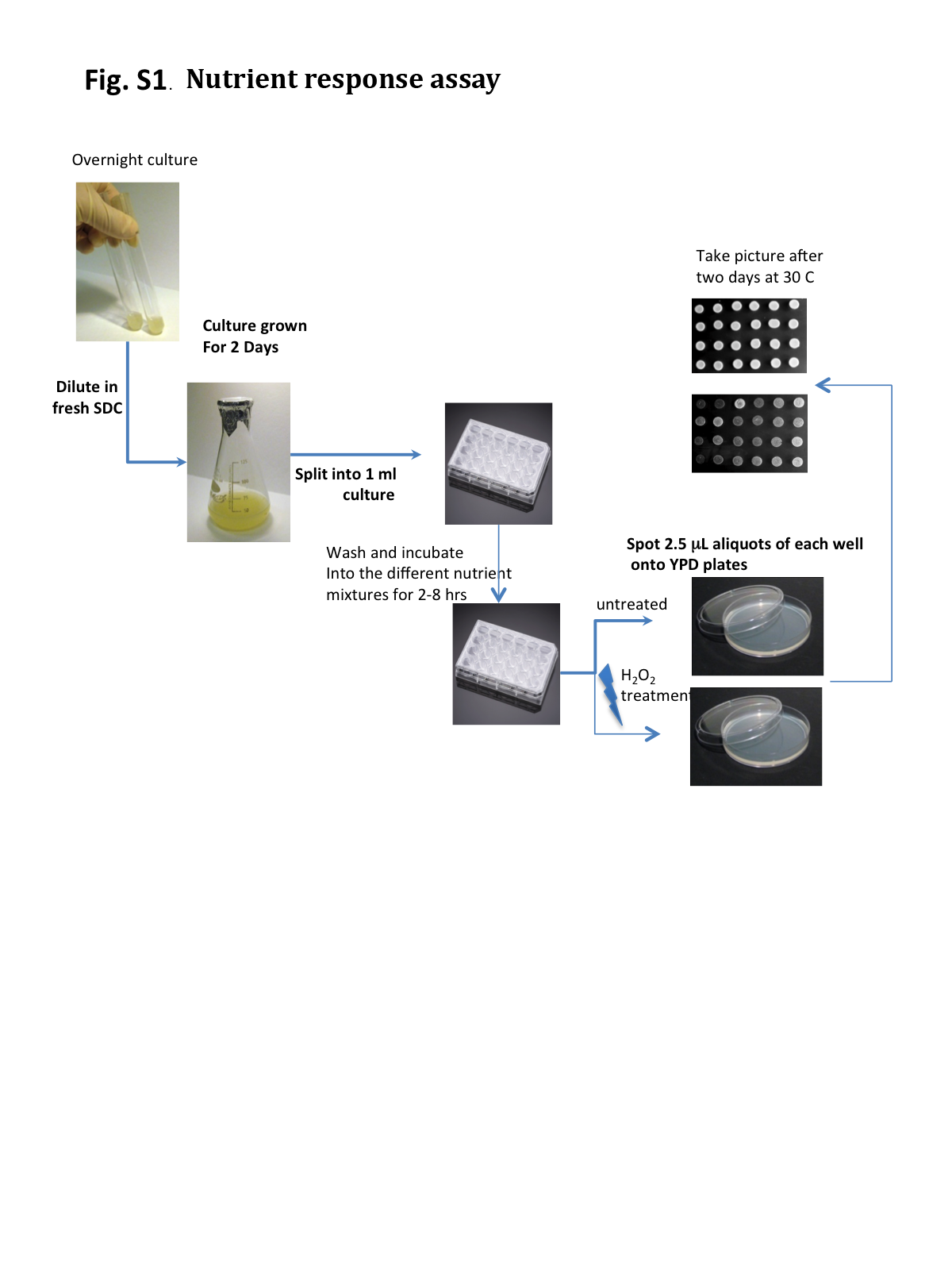

Supplement: Figure S1 — Nutrient response assay. Flowchart of the protocol used to assess the effect of single as well as mixture of nutrients addition on stress resistance at stationary phase. (TIF) [file pgen.1004113.s001.tif]

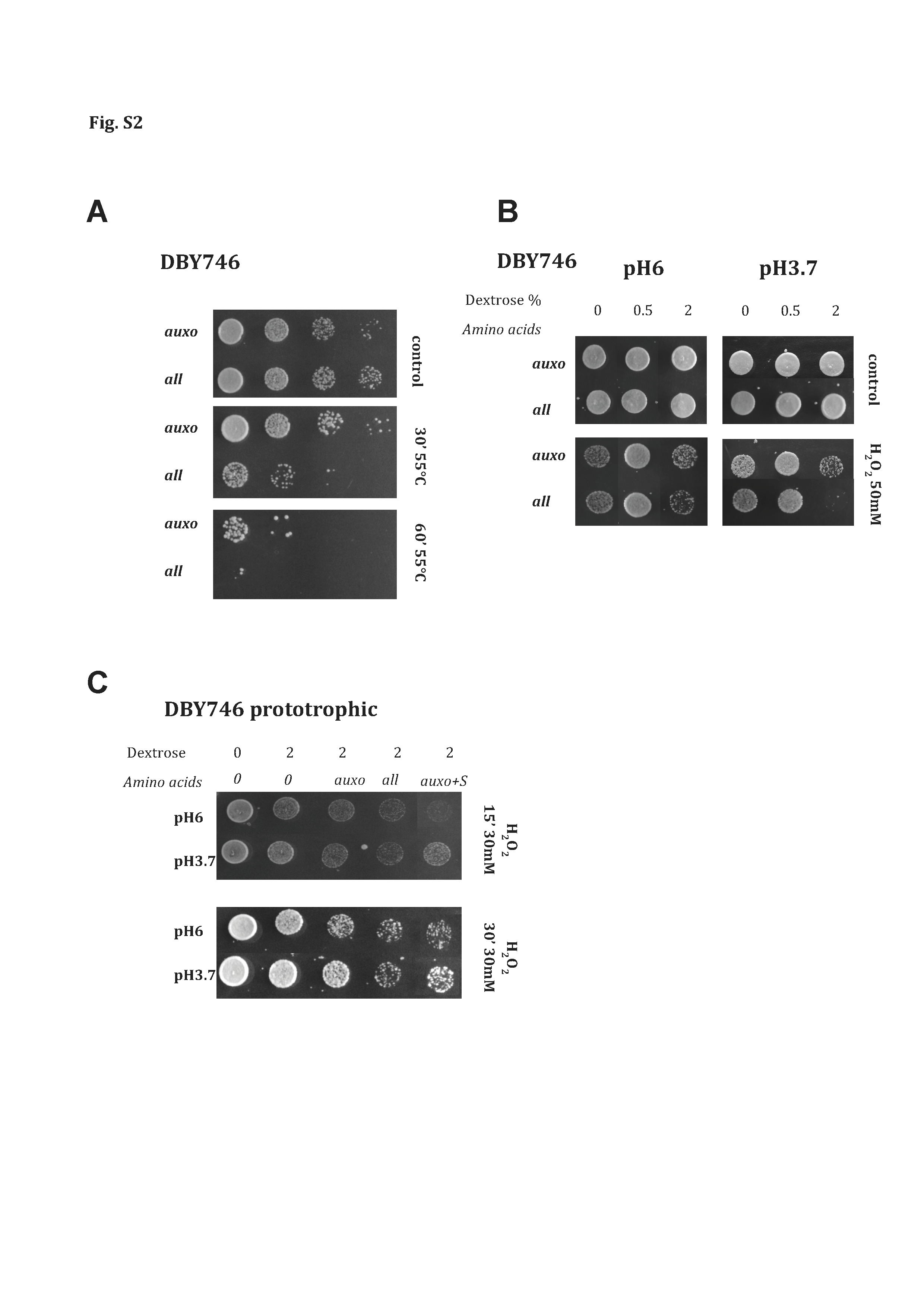

Supplement: Figure S2 — Acidification doesn't affect amino acid sensitivity. (A) Heat shock resistance of DBY746 wild type yeast strain grown to day 2 after a 4 hour pulse with a media containing 2% dextrose, all nitrogen source and either the amino acids necessary to compensate DBY746 auxotrophies (auxo) or the media with the complete mixture of amino acids (all). (B) Nutrient response assay in the presence of either the amino acids necessary to compensate DBY 746 auxotrophies (auxo) or the complete mixture of amino acids (all) at different percentages of dextrose at pH 6 and 3.7. (C) Nutrient response assay of prototrophic DBY746 yeast strain at pH 6 and 3.7 with different nutrient mixtures. Auxo indicates the addition of the compounds necessary to compensate the auxothrophies, all indicate the addition of the complete amino acid mixtures and auxo+S refers to the addition of the auxo mixture plus the amino acid serine at the standard concentration (for the list of the amino acid concentration used see table S2). (TIFF) [file pgen.1004113.s002.tiff]

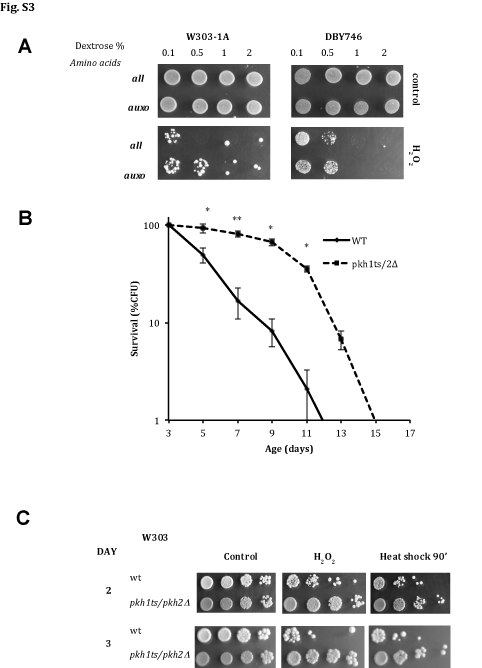

Supplement: Figure S3 — Dextrose concentration affects stress sensitivity in cooperation with amino acids in a Ras2-dependent fashion. (A) Nutrient response assay of two commonly used yeast wild type strains (w303-1A and DBY746) with increasing dextrose concentrations with the compounds necessary to compensate auxotrophies (auxo) or with the complete mixture of amino acids (all). Viability (B) of wild type W303 and of the indicated isogenic derivative. Viability from day 2 was switched to 35 C to inactivate the Pkh1-thermo sensitive allele. Stress (H2O2) and heat shock (55°C) resistance (C) of wild type W303 and pkh1ts/pkh2Δ isogenic derivative strains. (TIF) [file pgen.1004113.s003.tif]

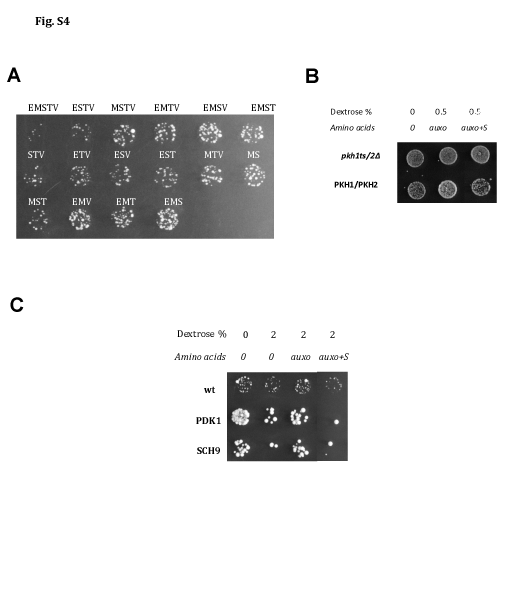

Supplement: Figure S4 — (A) Effect of amino acid mixtures on nutrient response. Amino acids are indicated with the single letter code. (B) Nutrient response assay of yeast cells expressing a thermo sensitive Pkh1 allele and a null Pkh2 allele and the corresponding strain co-transformed with Pkh1 and Pkh2 overexpression plasmids (PKH1/PKH2). (C) Nutrient response assay of DBY746 strain (wt), with the overexpression of mammalian ortholog of Pkhs (PDK1) or with the overexpression of Sch9 (SCH9). (TIF) [file pgen.1004113.s004.tif]

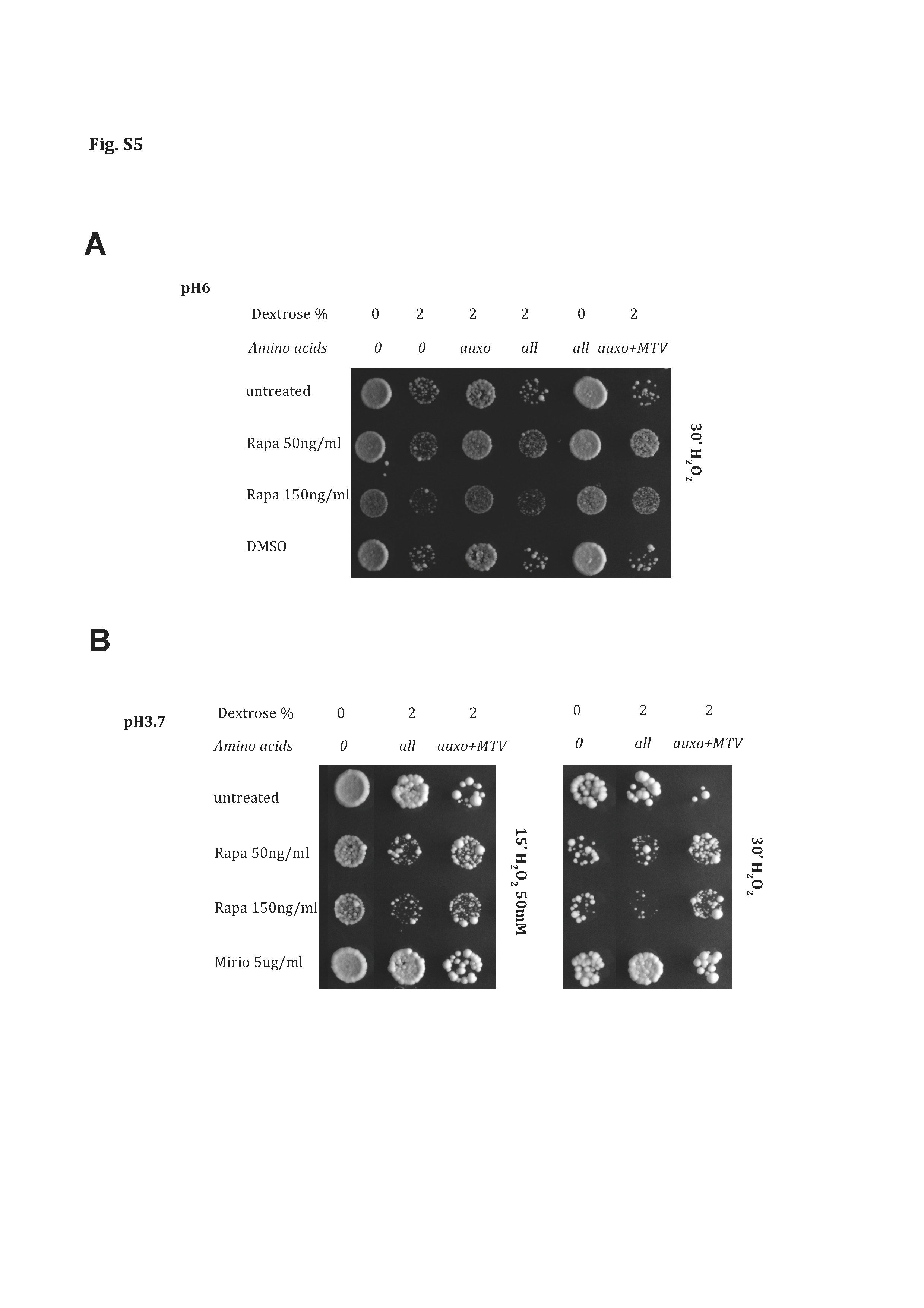

Supplement: Figure S5 — Rapamycin and myriocin treatments affect different amino acid signals. (A) Nutrient response assay in the presence of the Tor inhibitor Rapamicin (Rapa) at two different concentrations or with the same concentration of the solvent used to solubilize Rapamycin (DMSO). (B) Rapamycin versus Myriocin (Mirio) effect on nutrient response. (TIFF) [file pgen.1004113.s005.tiff]

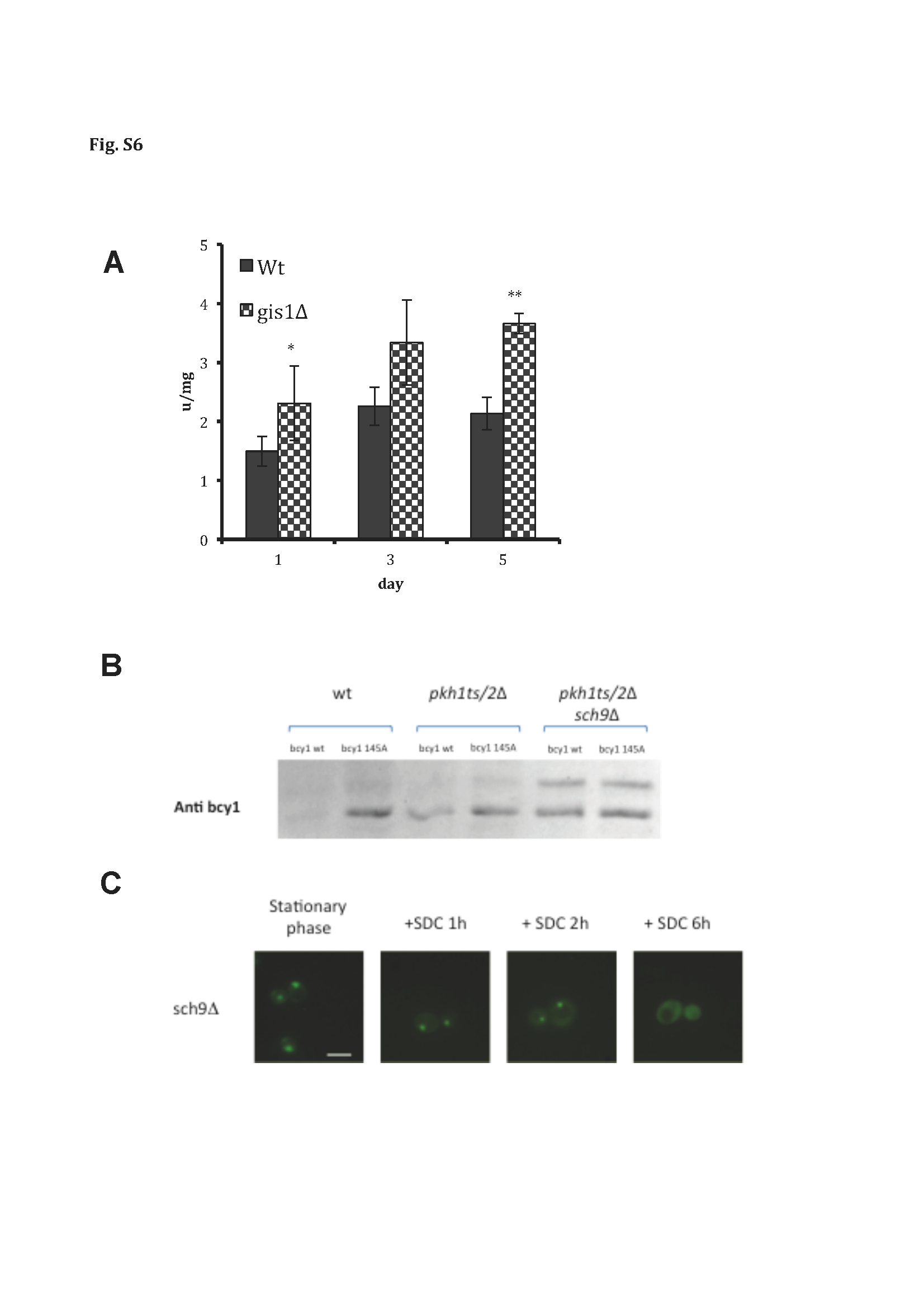

Supplement: Figure S6 — (A) STRE LacZ activity of the indicated isogenic strains. Experiments were performed in triplicate. Standard errors bars are shown. P values were evaluated by 2 tailed T-test for groups with unequal variants. * p = 0.1; **p<0.01. (B) Western blot using a commercially available anti Bcy1 antibody. Whole extract of the indicated strains in log phase (DBY746 genetic background) were used. (C) Fluorescence of sch9D strain carrying the Rim15-GFP fusion protein. Cells were grown to stationary phase, the exhausted medium was changed with fresh SDC medium, the fluorescence was measured after the indicated time. (TIFF) [file pgen.1004113.s006.tiff]
