# Supplementary material for: Serine- and Threonine/Valine-Dependent Activation of PDK and Tor Orthologs Converge on Sch9 to Promote Aging
Source: PLoS Genet. 2014 Feb 6;10(2):e1004113. doi: 10.1371/journal.pgen.1004113 (PMC3916422; doi:10.1371/journal.pgen.1004113)
Supplement: Table S1 — Yeast strains and plasmids used in this study. All the strains used in this study along with relevant genotype, reference and figure(s) where they appear are indicated. (DOC) [file pgen.1004113.s007.doc]

| **Strain** | **Genotype** | **Source** | **Figures** |
| --- | --- | --- | --- |
| DBY746  *ras2∆*  *sch9∆*  *sch9-HAtag*  *ras2∆sch9∆*  TS570  TS737  *pkh2Δ*  *pkh1^ts^/2Δ*  *gis1∆*  *msn2,4∆*  *msn2,4∆ gis1∆*  *rim15∆*  *Prototrophic*  *Wine yeast*  W303-1A | *MATα* *leu 2-3, 112 his3∆1 trp1-289 ura 3-52 GAL^+^*  DBY746 *ras2::LEU2*  DBY746 *sch9::URA3*  DBY746 SCH9HAtag::TRP1  DBY746 *ras2::LEU2 sch9::URA3*  DBY746 *SCH9*^T570A^  DBY746 *SCH9*^T737A^  DBY746 *pkh2*::LEU2  DBY746 *pkh1^ts::TRP1^ pkh2*::LEU2  DBY746 *gis1::HIS3*  DBY746 *msn2::HIS3 msn4::LEU2*  DBY746 *msn2::HIS3 msn4::LEU2 gis1::URA3*  DBY746 *rim15::LEU2*    *MAT*α *leu2-3,112 trp1-1 can1-100 ura3-1 ade2-1 his3-11,15*  W303-1A *pkh1^ts^ pkh2::TRP1*  W303-1A *sch9::URA3*  W303-1A SCH9-HAtag*::TRP1*  W303-1A *bcy1^S145A^*  W303-1A *pkh1^ts^ pkh2::TRP1 bcy1^S145A^*  W303-1A *sch9::URA3 bcy1^S145A^* | Gralla, 1991, J Bacteriol  Fabrizio, 2003, Genetics  Fabrizio, 2001, Science  This study  Wei, 2008  This study  This study  This study  This study  Wei, 2008  Fabrizio, 2001, Science  Wei, 2008, PLoS Genetics  Fabrizio, 2001, Science  Vini Corvo  This study  This study  This study  This study  This study | 1A-C, 2B-F, 3A-D, 4A-F, 5A-D, 6A-C, S2A-B, S3A, S4A, S4C, S5A-B, S6A  1C  1C, 2B-D, 2F, 5B, S6C  3E  1C  2B-D, 2F  2C-D, 2F  2E  2B, 2E  6A, S6A  6A  6A  6A-B  1A, 3E, S2C  1A  3E, S3A-C,S4C, S6B  S3B-C, S4B  S6B  3E  S6B  S6B  S6B |

**Table S1**. **Yeast strains and plasmids used in this study**

| **Plasmid** | **Description** | **Source** | **Figures** |
| --- | --- | --- | --- |
| pKT10PDK1  pRS416SCH9  pFD846  pRS416  pRS415  pAM202  pFR82  YEplac181PKH1 pKT10PKH2  pRP1661  pFA6a-3HA-TRP1  pYDF125TOR1ox | *URA3PDK1*  *URA3 SCH9*  *TRP1 RIM15-GFP*  *URA3*  *LEU2*  *LEU2 sch9T737A*  *LEU2 sch9T570A*  *PAB1-RFP-URA3* | Gift from Kevin A Morano  Gift from Claudio De Virgilio  Roelants FM et al, 2004, Microbiology  *«*  Gift from F. Luca  Longtine et al, 1998, Yeast | S4C  S4C  5A-C, S6C  S4C  4F  2C-D, 2F  2B-D, 2F  S4B  S4B  5C  3E  4F |
