# Supplementary material for: Serine- and Threonine/Valine-Dependent Activation of PDK and Tor Orthologs Converge on Sch9 to Promote Aging
Source: PLoS Genet. 2014 Feb 6;10(2):e1004113. doi: 10.1371/journal.pgen.1004113 (PMC3916422; doi:10.1371/journal.pgen.1004113)
Supplement: Table S2 — Composition of the medium used in all experiments except where otherwise specified. Synthetic complete glucose medium, SDC was modified with 4-fold excess of histidine, leucine, tryptophan, and uracil to compensate the auxotrophy of the DBY746 strain. pH was adjusted to 6.0 with NaOH. (DOCX) [file pgen.1004113.s008.docx]

**Table S2.**

| **Component** | **g/L** |
| --- | --- |
| D-glucose | 20 |
| Ammonium sulfate | 5 |
| **Table S2**  Yeast Nitrogen base (w/o amonium sulfate, w/o amino acids) | 1.8 |
| NaH2PO4 | 1.4 |
|  | **mg/L** |
| Adenine | 80 |
| L-Arginine | 40 |
| L-Aspartic acid | 100 |
| L-Glutamic acid | 100 |
| L-Histidine | 80 |
| L-Isoleucine | 60 |
| L-Leucine | 120 |
| L-Lysine | 60 |
| L-Methionine | 80 |
| L-Phenylalanine | 60 |
| L-Serine | 400 |
| L-Threonine | 200 |
| L-Tryptophan | 80 |
| L-Tyrosine | 40 |
| L-Valine | 150 |
| Uracil | 80 |
